# Bayesian inference for dependent stress–strength reliability of series–parallel system based on copula

**DOI:** 10.1038/s41598-025-13878-4

**Published:** 2025-08-12

**Authors:** Li Zhang, Rongfang Yan, Junrui Wang

**Affiliations:** 1 College of Mathematics and Statistics, Northwest Normal University, Lanzhou, 730070 China; 2 Gansu Provincial Research Center for Basic Disciplines of Mathematics and Statistics, Lanzhou, China

**Keywords:** Dependent stress-strength reliability, Series-parallel system, Clayton copula, Maximum likelihood estimation, Bayesian estimation, Scientific data, Statistics

## Abstract

In this paper, we investigate the inferential procedures for dependent stress-strength reliability within a series-parallel system, utilizing the Clayton copula to characterize the dependence structure between stress and strength variables, which follow proportional reversed hazard rate model. We establish maximum likelihood estimations for model parameters and system reliability, along with improved approximate confidence intervals based on Fisher information. Bayesian estimations are performed using the highly flexible Gamma-Beta prior distribution under different loss functions, and the highest posterior density interval is obtained via the Metropolis-Hastings algorithm. To assess the performance of the proposed methods, Monte Carlo simulations are conducted. Finally, an original data set, the general dam occupancy rate of Istanbul, is analyzed for illustrative purposes.

## Introduction

In the context of reliability engineering, the system’s reliability refers to the probability that it will perform its intended function effectively within a designated time frame and under specified environmental conditions. In reliability theory, the stress-strength model, initially introduced by Birnbaum^1^, plays an important role in reliability analysis and quality control. This model can assess the reliability of a system that has a random strength variable *X* and is subject to a random stress variable *Y*. The reliability, defined by the probability of the strength *X* exceeds the stress *Y*, i.e., $R=P(X>Y)$. As described by Kotz et al.^2^, the stress-strength model has been widely applied in various fields, including oceanography, hydrology, economics, and medicine. For example, if *X* denotes the strength of the rocket chamber and *Y* represents the maximum chamber pressure generated by the ignition of a solid propellant, then the reliability *R* can be defined as the probability of a successful engine firing. Several studies in the literature have explored the estimation of stress-strength reliability using various probability distributions, including Weibull, generalized Pareto, Burr XII, Kumaraswamy, Lindley, and Bathtub-shaped distributions. Noteworthy recent contributions on this topic include works by^3-9^.

The systems discussed in previous studies are predominantly restricted to single-component systems, which limits their applicability across various contexts. As science, technology, and manufacturing techniques advance, we always encounter numerous multicomponent systems in daily lives, including IT hardware, aero engines, keyboards, etc. And thus the investigation of multicomponent system reliability on stress-strength models has significant value and provides meaningful insights. Recently, several authors have contributed to the study of multicomponent stress-strength reliability. Eryılmaz and İşçioğlu^10^ investigated stress-strength reliability within the context of multicomponent multi-state systems modeling. Rao et al.^11^ explored the reliability inference of *k*-out-of-*n* systems for multicomponent stress-strength models under the assumptions of the Burr-XII distribution. Liu et al.^12^ derived the reliability estimation of multicomponent systems, accounting for the stress-strength model and specific N-M-cold-standby arrangements. Kohansal^13^ focused on estimating system reliability of a *k*-out-of-*n* system within a multicomponent stress-strength model, and using progressively censored Kumaraswamy samples. Kayal et al.^14^ obtained the point and interval estimates of multicomponent stress-strength reliability model of an *s*-out-of-*j* system using classical and Bayesian approaches by assuming both stress and strength variables follow the Chen distribution with a common shape parameter, which may be known or unknown. Saini et al.^15^ studied the classical and Bayesian estimation of multicomponent stress-strength reliability for *k*-out-of-*n* system based on progressively first-failure censored samples, where both stress and strength random variables follow the Burr XII distribution with a common first shape parameter. For more research on the estimation of multicomponent stress-strength reliability, one can refer to^16-19^.

In many research studies, it has been commonly assumed that stress and strength variables in reliability analysis are independent. However, strength and stress variables are dependent because they are often influenced by shared or common factors, such as material properties and environmental conditions. Recognizing this dependence is crucial, as it can significantly affect reliability, prompting some researchers to explore this issue within the context of dependent variables. Domma and Giordano^20^ evaluated stress-strength reliability by modeling the dependence through the FGM copula, considering Burr III distributions for both stress and strength. Gao et al.^21^ conducted a comprehensive evaluation of reliability, considering a mixed copula dependence structure between stress and strength variables by combining Frank, Clayton, and Gumbel copulas through a linear combination. Bai et al.^22^ investigated the estimation of stress-strength reliability when stress and strength were dependent through a Gumbel copula. Zhu^23^ derived the reliability estimation for a dependent multicomponent stress-strength model under the assumption of Clayton copula dependence among the variables.

**Fig. 1:**
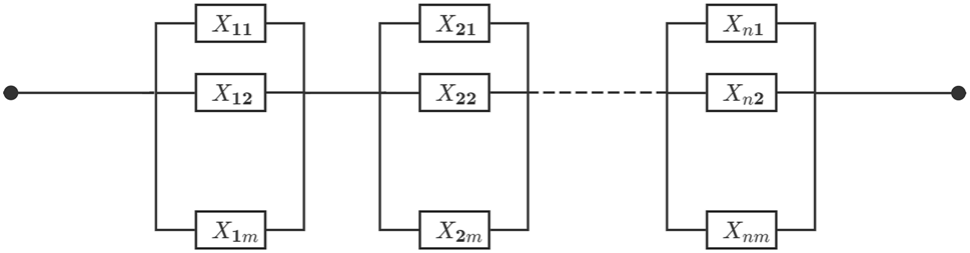
Series-parallel system.

The above research on dependent stress-strength reliability primarily focuses on series systems and k-out-of-n systems. In contrast, series-parallel systems offer significant advantages, such as enhanced reliability, fault tolerance, and flexibility, making them essential in high-performance applications, including power systems, communication networks, and aerospace equipment. For instance, in medical diagnostic devices such as MRI and CT scanners, various modules responsible for imaging, data processing, and thermal management are often configured redundantly to prevent system-wide failure. In automated manufacturing systems, production lines are typically arranged in a series-parallel architecture to ensure that localized malfunctions do not disrupt the entire workflow. Likewise, in transportation infrastructures–including railway networks and traffic signal control systems–series-parallel configurations provide enhanced route flexibility and maintain operational continuity in the presence of partial failures. To facilitate a better understanding of this system, Fig. 1 illustrates a representative structure of a series-parallel system. Recent studies, including Sadeghi et al.^24^, Zhang et al.^25^, and Gong et al.^26^, highlight the importance of series-parallel configurations and their applications across various fields. To our knowledge, there is a notable absence of research on the reliability of dependent stress-strength models within series-parallel systems. Therefore, this paper aims to provide reliability inference for dependent stress-strength models in series-parallel systems, where the stress and strength variables are dependent and follow a proportional reversed hazard rate model. We employ Clayton copula to effectively capture the dependence between stress and strength. Both classical and Bayesian statistical inference methods are utilized to estimate model parameters and system reliability.

The rest of this paper is organized as follows. In Section “Preliminaries”, we introduce basic concepts and model descriptions and derive the reliability of the series-parallel system based on some basic assumptions. The method-of-moment for the dependence parameter and the estimations for model parameters and *R* are presented in Section "Inference of model parameters and reliability". In this section, the MLE and Bayesian estimation of *R* are obtained, along with the corresponding confidence and credible intervals. A simulation study is performed to compare the effectiveness of the proposed estimations in Section “Simulation study”. In Section "Real data analysis", we analyze a real data set for illustrative purposes. Finally, we conclude the paper in Section “Conclusion”.

## Preliminaries

Before proceeding to the main results, let us first recall some basic concepts that will be used in the sequel.

### PRHR model

The random variable *T* with cumulative distribution function (CDF) *G*(*t*), probability density function (PDF) *g*(*t*), and reserved hazard rate function *r*(*t*) is said to follow the proportional reversed hazard rate (PRHR) model if

$$\begin{aligned} G(t)=F^\alpha (t), \quad g(t)=\alpha f(t)F^{\alpha -1} (t), \quad r(t) = \alpha r_{0}(t), \end{aligned}$$

where $\alpha$ is proportional parameter, *F*(*t*) is the baseline distribution, *f*(*t*) is the baseline PDF and $r_{0}(t) = f(t)/F(t)$ is the baseline reserved hazard rate function. The PRHR model offers significant advantages in reliability analysis due to its flexibility in capturing the varying risk profiles and failure mechanisms. Some well-known distributions such as power normal distribution, generalized exponential distribution, and exponentiated Weibull distribution are all special cases of the PRHR model. For more detailed applications of the PRHR model, one may refer to^27-29^.

### Copula

Copula is used to describe the dependence among random variables and construct joint distribution through marginal distributions. For a random vector $\varvec{X}=(X_1,X_2,\dots ,X_n)$ with joint $\operatorname {CDF} \ H$ and univariate marginal $\operatorname {CDFs} \ F_{1},F_{2},\dots ,F_{n}$, if there exists a function $C:[0,1]^n\rightarrow [0,1]$, such that, for all $x_{i}, i=1, 2,\dots ,n$,

$$\begin{aligned} H(\varvec{x})=C(F_1(x_1),F_2(x_2),\dots ,F_{n}(x_n)), \end{aligned}$$

then *C* is called the copula of random vector $\varvec{X}$.

Let *c* and *h* be the corresponding joint PDF of *C* and *H*, respectively, and $f_{i}$ be the corresponding PDF of $F_{i}, i= 1, 2,\dots ,n$, we have

1 $$\begin{aligned} h(x_{1},x_{2},\dots ,x_{n})= & \frac{\partial ^n H(x_{1},x_{2},\dots ,x_{n})}{\partial x_{1}\partial x_{2}\dots \partial x_{n}} \nonumber \\ = & \frac{\partial ^n C(F_{1}(x_{1}),F_{2}(x_{2}),\dots ,F_{n}(x_{n}))}{\partial F_{1}(x_{1})\partial F_{2}(x_{2})\dots \partial F_{n}(x_{n})} \prod _{i=1}^{n} f_{i}(x_{i}) \nonumber \\ = & c(F_{1}(x_{1}),F_{2}(x_{2}),\dots ,F_{n}(x_{n})) \prod _{i=1}^{n} f_{i}(x_{i}). \end{aligned}$$

Denote $u_{i}=F_{i}(x_{i}),\ i=1, 2,\dots ,n$, Copulas have many nice properties:

(i) If at least one $u_{i}, i=1, 2,\dots ,n$ is equal to zero, then

$$\begin{aligned} C(u_{1},\dots ,u_{i-1},0,u_{i+1},\dots ,u_{n})=0. \end{aligned}$$

(ii) If all $u_{i}, i=1, 2,\dots ,n$ are equal to 1 except $u_{i}$, then

$$\begin{aligned} C(1,\dots ,1,u_{i},1,\ldots ,1)=u_{i}. \end{aligned}$$

Archimedean copulas are rather popular because of the mathematical tractability and the capability of capturing a wide range of dependence. For a completely monotonic function $\psi :[0,+\infty )\mapsto [0,1]$, such that $\psi (0)=1$, $\psi (+\infty )=0$ and $(-1)^k \frac{\text {d}^{k}\psi (t)}{\text {d}t^{k}}\ge 0$. Then

$$\begin{aligned} C_\psi (u_1,\dots ,u_n)=\psi \big (\phi (u_1)+ \dots +\phi (u_n)\big ), \quad u_i\in [0,1], \quad i=1, 2,\dots ,n \end{aligned}$$

is said to be an Archimedean copula with generator $\psi$, where $\phi =\psi ^{-1}$ is the pseudo-inverse of $\psi$.

It is well known that the Archimedean family contains a great many useful copulas, including the well-known independence (product) copula, Clayton copula, Gumbel copula and Frank copula. A comprehensive exploration of copulas and their applications can be found in Nelsen^30^.

In this paper, we use the 2-dimensional strict Clayton copula with generator $\psi (t) =\frac{t^{-\theta } -1}{\theta }$ to depict the dependence structure between the stress and strength, which is given as

2 $$\begin{aligned} C_{\theta }(u, v)=\left( u^{-\theta }+v^{-\theta }-1\right) ^{-\frac{1}{\theta }}, ~~\theta \in (0,+\infty ), \end{aligned}$$

and the joint PDF of 2-dimensional strict Clayton copula can be derived as

3 $$\begin{aligned} c_{\theta }(u, v)=(\theta +1) \left( u^{-\theta }+v^{-\theta }-1\right) ^{-\frac{1}{\theta }-2}u^{-\theta -1} v^{-\theta -1},~~ \theta \in (0,+\infty ). \end{aligned}$$

Let $(x_{i}, y_{i})$($i=1, 2,\dots ,n$) and $(x_{j}, y_{j})$($j=1, 2,\dots ,n$) denote two observations of variable (*X*, *Y*), then we say that $(x_{i}, y_{i})$ and $(x_{j}, y_{j})$ are concordant if $(x_{i}-x_{j})(y_{i} - y_{j})>0$ and discordant if $(x_{i}-x_{j})(y_{i} - y_{j})<0$.

#### Definition 1

Let (*X*, *Y*) and $(X', Y')$ be independent and identically distributed random vectors, the Kendall’s tau of (*X*, *Y*) and $(X', Y')$ defined as

$$\begin{aligned} \tau =P[(X-X')(Y - Y')>0]-P[(X-X')(Y - Y')<0]. \end{aligned}$$

The Kendall’s tau can be used to quantify the concordant relationship between *X* and *Y*. If *X* and *Y* be continuous random variables whose copula is *C*. Then the population version of Kendall’s tau for *X* and *Y* is given by

4 $$\begin{aligned} \tau =4\int _{0}^{1}\int _{0}^{1}C(u, v)\mathrm dC(u, v)-1. \end{aligned}$$

In particular, if *C* is an Archimedean copula, Eq. (4) can be reformulated as follow

5 $$\begin{aligned} \tau =4\int _{0}^{1}\frac{\psi (t)}{\psi '(t)}\mathrm dt+1, \end{aligned}$$

where $\psi$ is the generator of the Archimedean copula.

### Model description

Consider an *m*-*n* series-parallel system consisting of *n* disjoint modules connected in series. Module *i* $(1\le i\le n)$ consisting of *m* components connected in parallel. Let $X_{ij} (i=1,2,\dots ,n; j=1,2, \ldots , m)$ denote a set of independent and identically distributed random variables with common CDF $F_{X}(x)$, representing the strength of components in the *m*-*n* series-parallel system. Additionally, let *Y* be a random variable representing the common stress, and it has a CDF $G_{Y}(y)$. Suppose that the stress variable *Y* is dependent on each $X_{ij}$. Denoting the strength of the system by *Z*, then the CDF of *Z* is given by

6 $$\begin{aligned} F_{Z}(z)=1-[1-F_{X}^m(z)]^n, \end{aligned}$$

and its PDF can be written as

7 $$\begin{aligned}&f_{Z}(z)=mn[1-F_{X}^m(z)]^{n-1}F_{X}^{m-1}(z)f_{X}(z). \end{aligned}$$

### Basic assumptions

**A1.** Each random strength variable $X_{ij}$ follows the PRHR model with baseline distribution *F* and proportional parameter $\alpha$. The CDF and PDF of $X_{ij}$ are given as

$$\begin{aligned} F_{X}(x \mid \alpha )=F^\alpha (x),\ \ \ x>0, ~~\alpha>0, \end{aligned}$$

and

$$\begin{aligned} f_{X}(x \mid \alpha )=\alpha f(x)F^{\alpha -1} (x)=\alpha r_{0}(x) F^\alpha (x),~~ x>0,~~ \alpha>0, \end{aligned}$$

respectively. The random stress variable *Y* follows the PRHR model with baseline distribution *F* and proportional parameter $\beta$. The CDF and PDF of *Y* are

$$\begin{aligned} G_{Y}(y \mid \beta )=F^\beta (y), ~~ y>0, ~~\beta>0, \end{aligned}$$

and

$$\begin{aligned} g_{Y}(y \mid \beta )=\beta f(y)F^{\beta -1} (x)=\beta r_{0}(y) F^\beta (y), ~~ y>0, ~~\beta>0, \end{aligned}$$

respectively. Hence, (6) and (7) can be rewritten as

$$\begin{aligned} F_{Z}(z\mid \alpha )=1-[1-F^{\alpha m}(z)]^n, \ \ \ z>0, ~~ \alpha>0, \end{aligned}$$

and

$$\begin{aligned} f_{Z}(z\mid \alpha )=mn\alpha r_{0}(z) F^{\alpha m}(z) [1-F^{\alpha m}(z)]^{n-1} , \ \ \ z>0, ~~\alpha>0, \end{aligned}$$

respectively.

**A2.** The dependence structure of the system strength *Z* and stress *Y* is modeled by 2-dimensional Clayton copula $C_{\theta }(u, v)$.

Based on the above assumptions, the reliability *R* of the system can be expressed as

8 $$\begin{aligned} R&= P\left( Z> Y\right) \nonumber \\ &= \int _{0}^{+\infty } \int _{0}^{z} \frac{\partial ^{2} C_{\theta }(u, v)}{\partial u \partial v} \bigg |_{\begin{array}{c} u=F_{Z}(z) \\ v=G(y) \end{array}} f_{Z}(z) g(y) \, \mathrm dy \mathrm dz \nonumber \\ &= \int _{0}^{+\infty } \frac{\partial C_{\theta }(u, v)}{\partial u} \bigg |_{\begin{array}{c} u=F_{Z}(z) \\ v=G(z) \end{array}} f_{Z}(z) \, \mathrm dz \nonumber \\ &= mn\alpha \int _{0}^{+\infty } r_{0}(z) F^{\alpha m}(z) [1 - F^{\alpha m}(z)]^{n - 1} \nonumber \\ &\quad \times \left( 1 - \left( 1 - F^{\alpha m}(z)\right) ^{n}\right) ^{-\theta - 1} \left( \left( 1 - \left( 1 - F^{\alpha m}(z)\right) ^{n}\right) ^{-\theta } + F^{-\beta \theta }(z) - 1\right) ^{-\frac{\theta + 1}{\theta }} \, \mathrm dz. \end{aligned}$$

## Inference of model parameters and reliability

### Method-of-moment for dependence parameter

In this subsection, a semi-parametric technique method-of-moment is used to estimate the dependence parameter $\theta$ based on the inversion of Kendall’s tau. This method is mostly used to the bivariate one-parameter case, but sometimes it also can be employed in the multivariate multiparameter cases. For more information on this method, one may refer to Oakes^31^; Genest and Rivest^32^; Kojadinovic and Yan^33^.

Denote $\varvec{X}_{i} = (Z_{i}, Y_{i}), i = 1, 2,\dots ,k$ the samples of 2-dimensional random variate $\varvec{X} = (\varvec{Z}, \varvec{Y})$ with sample size *k*. The method-of-moment approach relies on the inversion of a consistent estimator of a moment of the copula $C_{\theta }(u,v)$. Kendall’s tau, as described by Eq. (5), is one of the well-known moments commonly used in this method. Then the consistent estimator of the Kendall’s tau can be represented as

$$\begin{aligned} \hat{\tau _{k}}=\frac{4}{k(k-1)} \sum _{i \ne j} I_{(Z_{i} \le Z_{j})} I_{(Y_{i} \le Y_{j})}-1 . \end{aligned}$$

Considering that the Kendall’s tau of the Clayton copula is $\tau =\theta /(\theta +2)$, and given the one-to-one correspondence between $\tau$ and $\theta$, we can give the consistent estimator $\hat{\theta }$ of $\theta$ as follows

$$\begin{aligned} \hat{\theta }=\frac{2\hat{\tau _{k}}}{1-\hat{\tau _{k}}} . \end{aligned}$$

### Maximum likelihood estimation

In this subsection, we focus on the MLE of model parameters and *R*. Suppose that there are *k* systems subjected to a life-testing process, and the observed samples of the system strength and stress are $(z_{i}, y_{i})$, $i=1,2, \ldots , k$. Hence the likelihood function with observed samples $(y_{i},z_{i})$ is

$$\begin{aligned} & L(\alpha , \beta \mid \text{ data})\\ = & \prod _{i=1}^{k} c\left( F_{Z}(z_{i}),G(y_{i})\right) f_{Z}(z_{i}) g(y_{i}) \\ = & m^k n^k \alpha ^k \beta ^k \prod _{i=1}^{k} r_{0}(z_{i}) r_{0}(y_{i}) F^{\alpha m}(z_{i})F^{\beta }(y_{i})[1-F^{\alpha m}(z_{i})]^{n-1}c\left( 1-[1-F^{\alpha m}(z_{i})]^n,F^\beta (y_{i})\right) , \end{aligned}$$

and its log-likelihood function can be written as

$$\begin{aligned} l(\alpha , \beta \mid \text{ data})= & k\log m + k\log n + k\log \alpha + k\log \beta + \sum _{i=1}^{k} \log r_{0}(z_{i}) + \sum _{i=1}^{k} \log r_{0}(y_{i})\\ & + \alpha m \sum _{i=1}^{k} \log F(z_{i}) + \beta \sum _{i=1}^{k} \log F(y_{i}) + (n-1) \sum _{i=1}^{k} \log [1-F^{\alpha m}(z_{i})] \\ & +\sum _{i=1}^{k} \log c\left( 1-[1-F^{\alpha m}(z_{i})]^n,F^\beta (y_{i})\right) . \end{aligned}$$

For convenience, we denote

$$\begin{aligned} c_{u}(u,v)&= \frac{\partial c(u,v)}{\partial u}, \quad c_{v}(u,v)= \frac{\partial c(u,v)}{\partial v}, \quad \delta _{u}(u,v)= \frac{c_{u}(u,v)}{c(u,v)}, \quad \delta _{v}(u,v)= \frac{c_{v}(u,v)}{c(u,v)},\\ c_{uu}(u,v)&= \frac{\partial ^2 c(u,v)}{\partial u^2}, \quad c_{uv}(u,v)= \frac{\partial ^2 c(u,v)}{\partial u\partial v}, \quad c_{vv}(u,v)= \frac{\partial ^2 c(u,v)}{\partial v^2}, \quad \end{aligned}$$

and

$$\begin{aligned} \delta _{uu}(u,v)&= \frac{c_{uu}(u,v)}{c(u,v)}, \delta _{uv}(u,v)= \frac{c_{uv}(u,v)}{c(u,v)}, \quad \delta _{vv}(u,v)= \frac{c_{vv}(u,v)}{c(u,v)}, \end{aligned}$$

where $u= 1-[1-F^{\alpha m}(z)]^n,$ $v=F^\beta (y)$. By taking the first partial derivatives of $l(\alpha , \beta \mid \text{ data})$ with respect to $\alpha$ and $\beta$, and set to be zero, respectively, we have the following equations

9 $$\begin{aligned} \frac{\partial l(\alpha , \beta \mid \text{ data})}{\partial \alpha }&= \frac{k}{\alpha } - m(n-1)\sum _{i=1}^{k}\frac{F^{\alpha m}(z_{i})\log F(z_{i})}{1-F^{\alpha m}(z_{i})} \nonumber \\ &\ \ \ + m\sum _{i=1}^{k} \log F(z_{i}) \left[ 1+ n[1-F^{\alpha m}(z_{i})]^{n-1}F^{\alpha m}(z_{i}) \delta _{u_i}(u_{i}, v_{i})\right] =0, \end{aligned}$$

and

10 $$\begin{aligned} \frac{\partial l(\alpha , \beta \mid \text{ data})}{\partial \beta }&= \frac{k}{\beta } + \sum _{i=1}^{k} \log F(y_{i}) \left[ 1+ F^{\beta }(y_{i}) \delta _{v_i}(u_{i}, v_{i})\right] =0. \end{aligned}$$

Denote

11 $$\begin{aligned} J(\alpha ) =\dfrac{k}{ \begin{array}{c} - m\sum _{i=1}^{k} \log F(z_{i}) \left[ 1+ nF^{\alpha m}(z_{i})[1-F^{\alpha m}(z_{i})]^{n-1} \delta _{u_i}(u_{i}, v_{i})\right] \\ + m(n-1)\sum _{i=1}^{k}\frac{F^{\alpha m}(z_{i})\log F(z_{i})}{1-F^{\alpha m}(z_{i})} \end{array} }, \end{aligned}$$

and

12 $$\begin{aligned} H(\beta )=-\dfrac{k}{\sum _{i=1}^{k} \log F(y_{i}) \left[ 1+ F^{\beta }(y_{i}) \delta _{v_i}(u_{i}, v_{i})\right] }. \end{aligned}$$

Then, the MLEs of $\alpha$ and $\beta$ can be obtained by solving the nonlinear equations $J(\alpha )=\alpha$ and $H(\beta )=\beta$, respectively. It is difficult to solve the MLEs $\hat{\alpha }_{M}$ and $\hat{\beta }_{M}$ analytically from Eqs. (11) and (12), therefore, numerical methods, such as the Newton-Raphson iteration or other iterative techniques, necessitate consideration.

The following Theorem 1 affirms the existence on the MLEs of $\alpha$.

#### Theorem 1

The MLE of $\alpha$ exists.

#### Proof

From (9), it is easy to verify that $u_i= 1-[1-F^{\alpha m}(z_i)]^n \rightarrow 0$ and $F^{\alpha m}(z_i)\rightarrow 0$ when $\alpha \rightarrow \infty$. Note that

$$\begin{aligned} \lim _{\alpha \rightarrow \infty } -m(n-1) \sum _{i=1}^{k}\frac{F^{\alpha m}(z_{i})\log F(z_{i})}{1-F^{\alpha m}(z_{i})}=0, \end{aligned}$$

and

$$\begin{aligned} \lim _{\alpha \rightarrow \infty } F^{\alpha m}(z_{i}) \delta _{u_i}(u_{i}, v_{i})=\frac{1-(1-u_i)^{\frac{1}{n}}}{u_i} \frac{\theta v_{i}^{\theta } + u_{i}^{\theta }(v_{i}^{\theta }-1)(1 + \theta ) }{ v_{i}^{\theta }- u_{i}^{\theta }( v_{i}^{\theta }-1)}=\frac{\theta }{n}, \end{aligned}$$

then we have

$$\begin{aligned} \lim _{\alpha \rightarrow \infty } \frac{\partial l(\alpha , \beta \mid \text{ data})}{\partial \alpha } = m \sum _{i=1}^{k} \log F(z_i) (1 + \theta ) < 0. \end{aligned}$$

Note that $u_i= 1-[1-F^{\alpha m}(z_i)]^n \rightarrow 1$ and $F^{\alpha m}(z_i)\rightarrow 1$ when $\alpha \rightarrow 0$. For constant *c*, we have

$$\begin{aligned} \lim _{\alpha \rightarrow \infty } \delta _{u_i}(u_{i}, v_{i})=\theta v_{i}^{\theta } + (v_{i}^{\theta }-1)(1 + \theta )=c. \end{aligned}$$

Thus, we can obtain

$$\begin{aligned} \lim _{\alpha \rightarrow 0} \frac{\partial l(\alpha , \beta \mid \text{ data})}{\partial \alpha } = \infty + m \sum _{i=1}^{k} \log F(z_i)=\infty . \end{aligned}$$

Hence, the existence is proved. $\square$

#### Remark 1

**Fig. 2:**
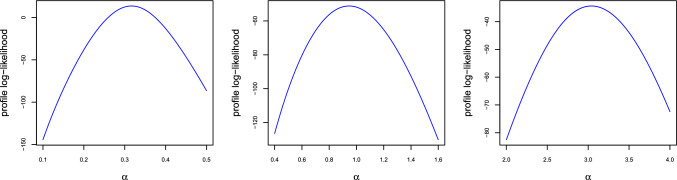
The profile log-likelihood plots of $\alpha$.

Figure 2 demonstrates that the profile log-likelihood function of $\alpha$ has a single peak, indicating the uniqueness of the MLE of $\alpha$. Although we cannot provide a rigorous theoretical proof, we present this as an open problem for further investigation by interested researchers. The parameters for generating the plots in Fig. 2 are as follows: $(m, n) = (2,2)$, $\beta = 1$, $\theta = 3$, and the base distribution $F(t) = 1 - \exp (-1.1t^{1.5})$. The values of $\alpha$ used for the three plots are 0.3, 1, and 3, respectively.

The next Theorem 2 gives the existence and uniqueness on the MLE of $\beta$.

#### Theorem 2

The MLE of $\beta$ not only exists but also remains unique.

#### Proof

From (10), it is easy to verify that $v_i=F^\beta (y_i)\rightarrow 0$ when $\beta \rightarrow \infty$. Note that

$$\begin{aligned} \lim _{\beta \rightarrow \infty } v_{i} \delta _{v_{i}}(u_{i}, v_{i})= \frac{\theta u_{i}^{\theta } + v_{i}^{\theta }(u_{i}^{\theta }-1)(1 + \theta ) }{ -v_{i}^{\theta } + u_{i}^{\theta }( v_{i}^{\theta }-1)}=-\frac{\theta u_{i}^{\theta }}{-u_{i}^{\theta }}=\theta , \end{aligned}$$

which leads to

$$\begin{aligned} \lim _{\beta \rightarrow \infty } \frac{\partial l(\alpha , \beta \mid \text{ data})}{\partial \beta } = \sum _{i=1}^{k} \log F(y_i) (1 + \theta ) < 0. \end{aligned}$$

Note that $v_i=F^\beta (y_i)\rightarrow 1$ as $\beta \rightarrow 0$, for constant *c*, we have

$$\begin{aligned} \lim _{\beta \rightarrow 0} \frac{\partial l(\alpha , \beta \mid \text{ data})}{\partial \beta } =c, \end{aligned}$$

which implies that

$$\begin{aligned} \lim _{\beta \rightarrow 0} \frac{\partial l(\alpha , \beta \mid \text{ data})}{\partial \beta } = \infty . \end{aligned}$$

Meanwhile,

$$\begin{aligned} \frac{\partial ^2 l(\alpha , \beta \mid \text{ data})}{\partial \beta ^2} = - \frac{k}{\beta ^2} + \sum _{i=1}^{k} \log F(y_i)\frac{\partial v_{i} \delta _{v_{i}}(u_{i}, v_{i})}{\partial v_i} \frac{\partial v_i}{\partial \beta } < 0, \end{aligned}$$

by noting the fact that

$$\begin{aligned} \frac{\partial v_{i} \delta _{v_{i}}(u_{i}, v_{i})}{\partial v_i}=\frac{\theta u_{i}^{\theta } v_{i}^{\theta -1}(u_{i}^{\theta }-1)(1 + 2\theta ) }{( v_{i}^{\theta } - u_{i}^{\theta }( v_{i}^{\theta }-1))^2}<0. \end{aligned}$$

Hence, both existence and uniqueness are proved. $\square$

Therefore, by substituting $\alpha , \beta$ for $\hat{\alpha }_{M}, \hat{\beta }_{M}$ into (8), the MLE $\hat{R}_{M}$ of *R* may be produced by applying the MLE’s invariance property.

### Approximate confidence intervals

In this subsection, we construct the approximate confidence intervals (ACIs) for parameters and reliability indices using the asymptotic normality of MLE. The Fisher information matrix of $(\alpha , \beta )$ is given by

$$\begin{aligned} I(\alpha , \beta )=\left[ \begin{array}{lll} I_{11} & I_{12} \\ I_{21} & I_{22} \end{array}\right] , \end{aligned}$$

where the elements of the matrix are derived by calculating the negative second partial derivatives of the log-likelihood function $l(\alpha , \beta \mid \text{ data})$, i.e.,

$$\begin{aligned} I_{11}&=-\frac{\partial ^{2} l(\alpha , \beta \mid \text{ data})}{\partial \alpha ^{2}}\\&= \frac{k}{\alpha ^2} + m^2(n-1)\sum _{i=1}^{k} \frac{F^{\alpha m}(z_{i})\log ^2 F(z_{i})}{\left( 1-F^{\alpha m}(z_{i})\right) ^2} - m^2n\sum _{i=1}^{k} \log ^2 F(z_{i})F^{\alpha m}(z_{i}) [1-F^{\alpha m}(z_{i})]^{n-2}\\ &\ \ \ \ \times \left[ [1-n F^{\alpha m}(z_{i})]\delta _u(u_{i},v_{i})+n F^{\alpha m}(z_{i}) [1-F^{\alpha m}(z_{i})]^n \left[ \delta _{uu}(u_{i},v_{i})-\delta _{u}^2(u_{i},v_{i})\right] \right] , \\ I_{12}&=I_{21}\\ &=-\frac{\partial ^{2} l(\alpha , \beta \mid \text{ data})}{\partial \alpha \partial \beta }\\ &= -mn\sum _{i=1}^{k} \log F(z_{i})\log F(y_{i})F^{\alpha m}(z_{i})F^{\beta }(y_{i}) [1-F^{\alpha m}(z_{i})]^{n-1}\left[ \delta _{uv}(u_{i},v_{i})-\delta _{u}(u_{i},v_{i})\delta _{v}(u_{i},v_{i})\right] , \end{aligned}$$

and

$$\begin{aligned} I_{22}=\frac{k}{\beta ^2} - \sum _{i=1}^{k} \log ^2 F(y_{i})F^{\beta }(y_{i}) \left[ \delta _v(u_{i},v_{i}) + F^{\beta }(y_{i})\left[ \delta _{vv}(u_{i},v_{i})-\delta _{v}^2(u_{i},v_{i})\right] \right] . \end{aligned}$$

Substituting the MLEs $\hat{\alpha }_{M}, \hat{\beta }_{M}$ for $\alpha , \beta$ into the matrix $I(\alpha , \beta )$ yields the observed Fisher information matrix. Thus, the approximate asymptotic variance-covariance matrix of parameters is obtained as follows

$$\begin{aligned} V(\alpha , \beta )=\left[ \begin{array}{lll} var(\hat{\alpha }) & cov(\hat{\alpha },\hat{\beta }) \\ cov(\hat{\beta }, \hat{\alpha }) & var(\hat{\beta }) \end{array}\right] . \end{aligned}$$

For $0 \le \gamma \le 1$, the $100(1 - \gamma )\%$ ACIs of $\alpha , \beta$ are given by

$$\begin{aligned} \left( \hat{\alpha }-z_{\gamma / 2} \sqrt{var(\hat{\alpha })},\;\; \hat{\alpha }+z_{\gamma / 2} \sqrt{var(\hat{\alpha })}\right) ,\\ \left( \hat{\beta }-z_{\gamma / 2} \sqrt{var(\hat{\beta })},\;\; \hat{\beta }+z_{\gamma / 2} \sqrt{var(\hat{\beta })}\right) , \end{aligned}$$

where $z_{\gamma / 2}$ is the $\gamma / 2$ percentile point of the standard normal distribution. However, sometimes the lower bound of this confidence interval may be less than 0, which contradicts with the prerequisite $\alpha , \beta> 0$. The delta method is employed here to further obtain better ACIs. For more details about delta method, please refer to Oehlert^34^. The approximate normal distribution of $\ln \alpha$ can be obtained by logarithmic transformation $\ln \hat{\alpha }\sim N\left( \ln \alpha , var(\ln \hat{\alpha })\right)$, where $var(\ln \hat{\alpha })=\frac{var(\hat{\alpha })}{\hat{\alpha }^2}$. Then the improved $100(1-\gamma ) \%$ ACIs of $\alpha$ is given by

$$\begin{aligned} \left( \hat{\alpha }\cdot \exp \left( -\frac{z_{\gamma /2}\sqrt{var(\hat{\alpha })}}{\hat{\alpha }}\right) ,\;\;\hat{\alpha }\cdot \exp \left( \frac{z_{\gamma / 2}\sqrt{var( \hat{\alpha })}}{\hat{\alpha }}\right) \right) . \end{aligned}$$

Similar to the above process, the improved $100(1-\gamma )\%$ ACIs of $\beta$ can be obtained

$$\begin{aligned} \left( \hat{\beta }\cdot \exp \left( -\frac{z_{\gamma /2}\sqrt{var(\hat{\beta })}}{\hat{\beta }}\right) ,\;\;\hat{\beta }\cdot \exp \left( \frac{z_{\gamma / 2}\sqrt{var( \hat{\beta })}}{\hat{\beta }}\right) \right) . \end{aligned}$$

In accordance with the asymptotic normality of MLE and the multivariate central limit theorem, when $k\rightarrow \infty$, we have

$$\begin{aligned} \left( ( \hat{\alpha },\hat{\beta })-(\alpha ,\beta )\right) \sim N\left( 0, V \right) . \end{aligned}$$

Define $G^{T} =\left( \frac{\partial R}{\partial \alpha },\frac{\partial R}{\partial \beta }\right)$, according to the multivariant Delta method, the asymptotic distribution of *R* is given as

$$\begin{aligned} ( \hat{R}-R) \sim N\left( 0, G^{T}VG \right) . \end{aligned}$$

Hence, the $100(1-\gamma )\%$ ACI for *R* can be written as

$$\begin{aligned} \left( \hat{R}-z_{\gamma / 2} \sqrt{G^{T}VG},\;\; \hat{R}+z_{\gamma / 2} \sqrt{G^{T}VG}\right) . \end{aligned}$$

Note that $0 \le R \le 1$, the $100(1 - \gamma )\%$ ACI of *R* should be rewritten by

$$\begin{aligned} \left( \max {\left\{ 0, \hat{R}-z_{\gamma / 2} \sqrt{G^{T}VG}\right\} },\;\; \min {\left\{ \hat{R}+z_{\gamma / 2} \sqrt{G^{T}VG},1 \right\} }\right) . \end{aligned}$$

### Bayesian estimation

Bayesian theory has received a lot of attention by statisticians as an effective alternative to the classical frequency school. The ability to integrate prior information in Bayesian analysis plays an important role in solving problems with limited data availability such as reliability analysis. In this study, our focus lies in the reliability analysis of stress strength dependence. Therefore, a suitable prior distribution is used to characterize the dependence among model parameters.

Following the methodology proposed by Peña and Gupta^35^, to derive the dependent prior distribution of $\alpha$ and $\beta$, we define $\lambda = \alpha + \beta$. We then assume that the prior distribution of $\lambda$ is

$$\begin{aligned} \pi _{0}(\lambda \mid a,b)=\frac{b^{a}}{\Gamma (a)}\lambda ^{a-1}e^{-b\lambda }, \quad \lambda>0, ~~a, b>0. \end{aligned}$$

For a given $\lambda$, the prior distribution of $\frac{\alpha }{\lambda }$ is a conditional beta distribution,

$$\begin{aligned} \pi _{1}\left( \frac{\alpha }{\lambda }\Big | \lambda , c_{1},c_{2}\right) =\frac{\Gamma (c_{1}+c_{2})}{\Gamma (c_{1})\Gamma (c_{2})}\left( \frac{\alpha }{\lambda }\right) ^{c_{1}-1}\left( \frac{\beta }{\lambda }\right) ^{c_{2}-1}, \quad c_{1},c_{2}>0. \end{aligned}$$

Let $c=c_{1}+c_{2}$, then, the joint prior of $(\alpha ,\beta ,\lambda )$ can be written as

$$\begin{aligned} \pi _{2}\left( \frac{\alpha }{\lambda }, \lambda \big | a,b, c_{1},c_{2}\right) =\frac{\Gamma (c)}{\Gamma (a)} b^{a-c} \lambda ^{a-c+1} \frac{b^{c_{1}}}{\Gamma (c_{1})}\alpha ^{c_{1}-1}e^{-b\alpha } \frac{b^{c_{2}}}{\Gamma (c_{2})}\beta ^{c_{2}-1}e^{-b\beta }. \end{aligned}$$

Furthermore, by the Jacobian transformation, the joint prior of $(\alpha ,\beta )$ can be derived as

13 $$\begin{aligned} \pi _{3}\left( \alpha , \beta \mid a,b, c_{1},c_{2}\right) =\frac{\Gamma (c)}{\Gamma (a)} b^{a-c} \lambda ^{a-c} \frac{b^{c_{1}}}{\Gamma (c_{1})}\alpha ^{c_{1}-1}e^{-b\alpha } \frac{b^{c_{2}}}{\Gamma (c_{2})}\beta ^{c_{2}-1}e^{-b\beta }. \end{aligned}$$

This prior distribution is known as the Gamma-Beta (GB) distribution with hyperparameters $a,b, c_{1},c_{2}$. By selecting appropriate hyperparameter values, this type of prior distribution is highly flexible and can explain the various scenarios in which the parameters are independent or dependent.

Thus, the joint posterior distribution of $(\alpha ,\beta )$ can be formulated as

$$\begin{aligned} \pi ^{*}\left( \alpha , \beta \mid \text {data}\right)\propto & \alpha ^{k+c_{1}-1}\beta ^{k+c_{2}-1} e^{-b(\alpha +\beta )}(\alpha +\beta )^{a-c} \prod _{i=1}^{k} r(z_{i}) r(y_{i}) F^{\alpha m}(z_{i})F^{\beta }(y_{i})\\ & \times [1-F^{\alpha m}(z_{i})]^{n-1} c\left( 1-[1-F^{\alpha m}(z_{i})]^n,F^\beta (y_{i})\right) . \end{aligned}$$

And we further have

$$\begin{aligned} \log \pi ^{*}\left( \alpha , \beta \mid \text {data}\right)= & (k+c_{1}-1)\log \alpha + (k+c_{2}-1)\log \beta -b\alpha -b\beta +(a-c)\log (\alpha +\beta )\\ & + \sum _{i=1}^{k} \log r_{0}(z_{i}) + \sum _{i=1}^{k} \log r_{0}(y_{i}) + \alpha m \sum _{i=1}^{k} \log F(z_{i}) + \beta \sum _{i=1}^{k} \log F(y_{i}) \\ & +(n-1) \sum _{i=1}^{k} \log [1-F^{\alpha m}(z_{i})] +\sum _{i=1}^{k} \log c\left( 1-[1-F^{\alpha m}(z_{i})]^n,F^\beta (y_{i})\right) . \end{aligned}$$

Under the square error loss function (SEL), the linex loss function (LL), and the general entropy loss function (GEL), the Bayesian estimators are derived by minimizing the risk functions as follows

$$\begin{aligned}&\ \hat{\eta }_{SEL}= E(\eta \mid x),\\&\ \hat{\eta }_{LL}= -\frac{1}{q}\log E(e^{-q\eta }\mid x),\\&\ \hat{\eta }_{GEL}= \left( E(\eta ^{-w}\mid x)\right) ^{-\frac{1}{w}}, \end{aligned}$$

respectively, where *q* and *w* are predetermined constants, $\eta$ represents unknown parameters $\alpha , \beta$. It is difficult to obtain explicit solutions for these complex integrals through direct computation. To address these computational difficulties, Markov Chain Monte Carlo (MCMC) sampling techniques, specifically the Metropolis-Hastings (M-H) algorithm is performed for Bayesian estimators and the highest posterior density (HPD) credible intervals. The algorithm’s procedure can be summarized as follows (Algorithm 1).

**Algorithm 1:**
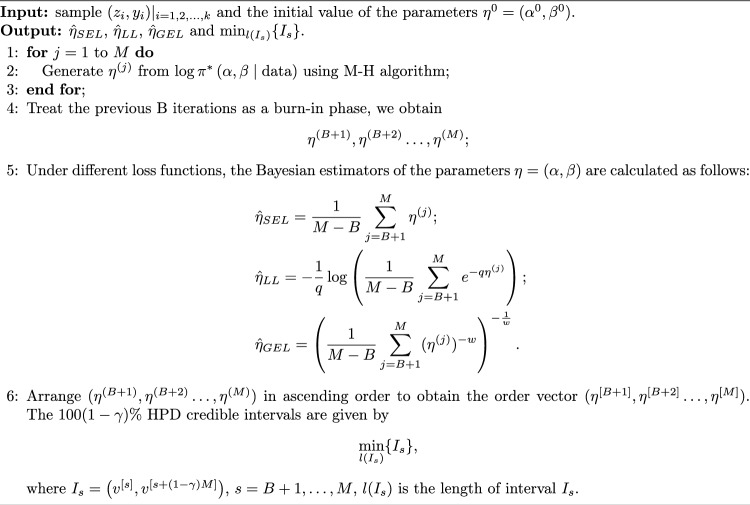
The algorithm of Bayesian estimators and HPD credible intervals

Thus, substituting $\hat{\eta }_{SEL},\hat{\eta }_{LL}$ and $\hat{\eta }_{GEL}$ into (8), the Bayesian estimates $\hat{R}_{SEL}, \hat{R}_{LL}$, and $\hat{R}_{GEL}$ of *R* can be obtained.

## Simulation study

Before conducting the simulation study, we clarify the choice of the baseline distribution. Although the proposed inference method is developed under the PRHR model without specifying any particular baseline distribution, the Weibull distribution with cumulative distribution function $F(t) = 1 - \exp (-\lambda _1 t^{\lambda _2})$ is adopted in the simulation study as a representative case. The Weibull distribution is a well-known member of the PRHR family, commonly used in practice due to its flexibility in modeling various failure behaviors. This choice facilitates interpretation and numerical validation of the proposed estimators without affecting the generality of our theoretical results. Based on this setup, the reliability *R* of the system can be expressed as

$$\begin{aligned} R&= mn\alpha \int _{0}^{+\infty } \lambda _1 \lambda _2 z^{\lambda _2 - 1} \exp (-\lambda _1 z^{\lambda _2}) \left( 1 - \exp (-\lambda _1 z^{\lambda _2})\right) ^{\alpha m-1} \nonumber \\&\quad \times \left[ 1 - \left( 1 - \exp (-\lambda _1 z^{\lambda _2})\right) ^{\alpha m}\right] ^{n - 1} \left( 1 - \left[ 1 - \left( 1 - \exp (-\lambda _1 z^{\lambda _2})\right) ^{\alpha m}\right] ^{n}\right) ^{-\theta - 1} \nonumber \\&\quad \times \left( \left( 1 - \left[ 1 - \left( 1 - \exp (-\lambda _1 z^{\lambda _2})\right) ^{\alpha m}\right] ^{n}\right) ^{-\theta } + \left( 1 - \exp (-\lambda _1 z^{\lambda _2})\right) ^{-\beta \theta } - 1 \right) ^{-\frac{\theta + 1}{\theta }} \, \textrm{d}z. \end{aligned}$$

Based on the above setup, we carry out a simulation study to evaluate the performances of the aforementioned estimates for the unknown parameters and reliability *R*. The performance of the point estimates will be compared based on average bias (AB), and mean square error (MSE). In addition, we compute average width (AW) and coverage probability (CP) for interval estimates. For the point estimation, MLE and Bayes estimates are reported, whereas, for the interval estimates, ACIs and HPD credible intervals are constructed. Suppose that the dependent stress-strength samples $(\varvec{z}, \varvec{y})$ derived from Clayton copula *C*(*u*, *v*), and denote $c_{u}(v)=\partial C(u, v)/\partial u$. In all simulation scenarios, the dependence parameter $\theta$ is estimated via the method-of-moment approach based on the inversion of Kendall’s tau, as introduced in subsection "Method-of-moment for dependence parameter". The following algorithm 2 is used to generate $(\varvec{z}, \varvec{y})$. All simulations were conducted in R (version 4.3.0) using the *copula* and *stats*4 packages.

**Algorithm 2:**
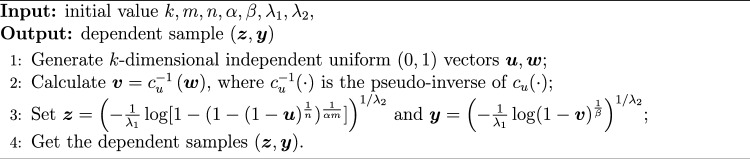
Generating the dependent stress-strength samples.

Let us consider a 2-2 parallel-series system in the multicomponent stress-strength model, i.e., $(m, n)=(2,2)$. Take $\alpha =0.8$, $\beta =0.7$, and choose the baseline distribution as $F(t)= 1- \exp (-1.1t^{1.5})$. For given dependence parameters $\theta =3$, $\theta =4$ and $\theta =5$, the respective true values of *R* are 0.681871, 0.699051 and 0.711488. For MLE, we employ the Newton-Raphson and Delta techniques to derive point estimators and interval estimations of model parameters and reliability, respectively. The entire process is replicated 10,000 times.

**Fig. 3:**
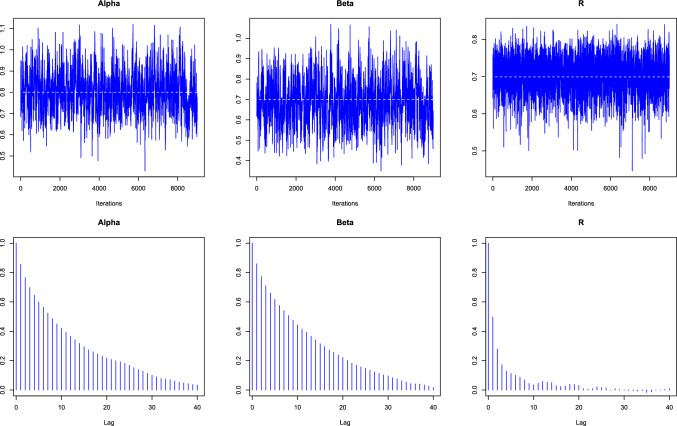
The trace and autocorrelation plots of $\alpha$, $\beta$ and *R* for MCMC chain.

The Bayesian estimations are performed under SEL, LL and GEL function and a nearly non-informative proper priors by setting hyperparameter values as $a = b = 0.001$, $c_{1}=c_{2}=1$ and $q= w= 0.1$. In order to eliminate the influence of the initial value, the MCMC algorithm is simulated 10,000 times, and the first 1000 times are used as annealing. It should be noted that MCMC output analysis is necessary for assessing the convergence of the iteration process in the M-H algorithm (Algorithm 1), and the analytical results are shown in Fig. 3.

The point estimation simulation results are shown in Tables 1,2,3, and the interval estimation simulation results are given in Tables 4,5,6. From Tables 1,2,3, we observe that the ABs and MSEs of the MLE and Bayesian estimation for the model parameters and reliability *R* exhibit a downward trend as the value of *k* increases. This trend indicates that the accuracy of the estimations improves with larger sample sizes. Notably, the Bayesian estimations are closer to the true values compared to those obtained through MLE. Specifically, the AB of the Bayesian estimation based on the GEL function is minimized. Given a specific sample size *k*, the model parameters and reliability *R* do not change significantly with the increase in the dependent parameter. These results show that the point estimation ${\textrm{Bayes}}_{GEL}$ has the best performance in terms of AB.

**Table 1:** The ABs and MSEs of MLEs and Bayesian estimations for $\alpha$, $\beta$ and *R* ($\theta =3$).

*k*	Para	MLE		${\textrm{Bayes}}_{\textrm{SEL}}$		${\textrm{Bayes}}_{\textrm{LL}}$		${\textrm{Bayes}}_{\textrm{GEL}}$	
		AB	MSE	AB	MSE	AB	MSE	AB	MSE
	$\alpha$	0.029151	0.013815	0.016570	0.012854	0.016011	0.012801	0.099146	0.012445
20	$\beta$	0.036544	0.018644	0.023096	0.017145	0.022372	0.017044	0.012327	0.016289
	*R*	−0.006506	0.001414	−0.005617	0.001402	−0.004657	0.001372	−0.002400	0.001337
	$\alpha$	0.012579	0.005756	0.006398	0.005603	0.006131	0.005593	0.002781	0.005522
40	$\beta$	0.013972	0.007127	0.007635	0.006923	0.007302	0.006904	0.002500	0.006773
	*R*	−0.001724	0.000581	−0.001556	0.000579	−0.001143	0.000574	−0.000082	0.000568
	$\alpha$	0.006698	0.003812	0.002640	0.008742	0.024466	0.003738	0.000263	0.003714
60	$\beta$	0.010153	0.004844	0.005926	0.004740	0.005708	0.004732	0.002546	0.004667
	*R*	−0.002374	0.000364	−0.002226	0.000363	−0.001957	0.000360	−0.001256	0.000356
	$\alpha$	0.004942	0.002596	0.001823	0.002552	0.001693	0.002549	0.000044	0.002537
80	$\beta$	0.006714	0.003346	0.003451	0.003278	0.003290	0.003274	0.000938	0.003244
	*R*	−0.001347	0.000279	−0.001216	0.000279	−0.001022	0.000277	−0.000504	0.000275

**Table 2:** The ABs and MSEs of MLEs and Bayesian estimations for $\alpha$, $\beta$ and *R* ($\theta =4$).

*k*	Para	MLE		${\textrm{Bayes}}_{\textrm{SEL}}$		${\textrm{Bayes}}_{\textrm{LL}}$		${\textrm{Bayes}}_{\textrm{GEL}}$	
		AB	MSE	AB	MSE	AB	MSE	AB	MSE
	$\alpha$	0.028547	0.012637	0.016020	0.011762	0.015496	0.011716	0.009049	0.011397
20	$\beta$	0.035212	0.014637	0.021876	0.015514	0.021428	0.015423	0.011831	0.014770
	*R*	−0.005460	0.001004	−0.004537	0.001039	−0.004119	0.001026	−0.003372	0.001040
	$\alpha$	0.011847	0.005272	0.005808	0.005135	0.005559	0.005138	0.004243	0.005247
40	$\beta$	0.013256	0.006553	0.007060	0.006373	0.006528	0.006326	0.005924	0.006487
	*R*	−0.001555	0.000412	−0.001162	0.000435	−0.000757	0.000458	−0.000441	0.000458
	$\alpha$	0.006753	0.003554	0.002671	0.003548	0.003481	0.003541	0.000442	0.003548
60	$\beta$	0.009784	0.004488	0.005508	0.004382	0.004375	0.004325	0.002360	0.004317
	*R*	−0.002105	0.000259	−0.001784	0.000282	−0.001663	0.000275	−0.000997	0.000253
	$\alpha$	0.004638	0.002411	0.001730	0.002377	0.001608	0.002375	0.000666	0.002364
80	$\beta$	0.006186	0.003052	0.003165	0.003000	0.002305	0.002952	0.001833	0.003014
	*R*	−0.001168	0.000196	−0.000954	0.000196	−0.000867	0.000195	−0.000377	0.000194

**Table 3:** The ABs and MSEs of MLEs and Bayesian estimations for $\alpha$, $\beta$ and *R* ($\theta =5$).

*k*	Para	MLE		$\text {Bayes}_{\textrm{SEL}}$		$\text {Bayes}_{\textrm{LL}}$		$\text {Bayes}_{\textrm{GEL}}$	
		AB	MSE	AB	MSE	AB	MSE	AB	MSE
	$\alpha$	0.028037	0.011798	0.015669	0.010970	0.015174	0.010929	0.009070	0.010637
20	$\beta$	0.034270	0.015627	0.021108	0.014373	0.020745	0.014296	0.016224	0.013705
	*R*	−0.005125	0.000779	−0.003839	0.001472	−0.003074	0.001471	−0.001622	0.001375
	$\alpha$	0.011256	0.004940	0.005332	0.004830	0.005096	0.004822	0.002134	0.004770
40	$\beta$	0.012707	0.006161	0.006631	0.006009	0.006339	0.005996	0.002125	0.005896
	*R*	−0.001472	0.000321	−0.000922	0.000334	−0.000889	0.000332	−0.000584	0.000341
	$\alpha$	0.006820	0.003372	0.002933	0.003340	0.002778	0.003336	0.000814	0.003314
60	$\beta$	0.009577	0.004240	0.005522	0.004178	0.005330	0.004172	0.002541	0.004118
	*R*	−0.001946	0.000203	−0.001536	0.000178	−0.001520	0.000172	−0.000876	0.000198
	$\alpha$	0.004375	0.002280	0.001576	0.002262	0.001460	0.002261	−0.000007	0.002251
80	$\beta$	0.005781	0.002866	0.002866	0.002824	0.002724	0.002821	0.000653	0.002798
	*R*	−0.001052	0.000152	−0.000758	0.000152	−0.000750	0.000151	−0.000275	0.000150

**Table 4:** The AWs and CPs of ACIs and HPDs for $\alpha$, $\beta$ and *R* ($\theta =3$).

*k*	Para	ACI				HPD			
		AW	CP	Lower	Upper	AW	CP	Lower	Upper
	$\alpha$	0.423311	0.932000	0.644098	1.067409	0.406002	0.935000	0.616967	1.022969
20	$\beta$	0.482074	0.929000	0.533992	1.016066	0.458281	0.936000	0.498455	0.956735
	*R*	0.130131	0.934000	0.610750	0.740881	0.156426	0.970000	0.597324	0.753751
	$\alpha$	0.290710	0.952000	0.680126	0.970836	0.282559	0.948000	0.666813	0.949372
40	$\beta$	0.325201	0.945000	0.569668	0.894869	0.314763	0.944000	0.552592	0.867355
	*R*	0.089547	0.950000	0.653373	0.742921	0.106170	0.970000	0.626847	0.733017
	$\alpha$	0.235477	0.946000	0.697508	0.932985	0.228580	0.938000	0.689392	0.917972
60	$\beta$	0.263479	0.950000	0.590536	0.854015	0.255328	0.937000	0.579617	0.834945
	*R*	0.073379	0.948000	0.642807	0.716187	0.086300	0.982000	0.636295	0.722595
	$\alpha$	0.203155	0.951000	0.709749	0.912904	0.197740	0.949000	0.703860	0.901600
80	$\beta$	0.226297	0.946000	0.602570	0.828867	0.219916	0.945000	0.594556	0.814472
	*R*	0.063032	0.953000	0.649008	0.712041	0.073888	0.972000	0.643552	0.714740

**Table 5:** The AWs and CPs of ACIs and HPDs for $\alpha$, $\beta$ and *R* ($\theta =4$).

*k*	Para	ACI				HPD			
		AW	CP	Lower	Upper	AW	CP	Lower	Upper
	$\alpha$	0.407125	0.930000	0.649644	1.056770	0.392705	0.941000	0.624232	1.015137
20	$\beta$	0.461500	0.927000	0.539869	1.001369	0.441955	0.938000	0.505133	0.947088
	*R*	0.109391	0.929000	0.638895	0.748287	0.132987	0.927000	0.627891	0.760878
	$\alpha$	0.279399	0.950000	0.684083	0.963483	0.272386	0.946000	0.671416	0.943801
40	$\beta$	0.311575	0.948000	0.574299	0.885874	0.302655	0.949000	0.557934	0.860589
	*R*	0.075171	0.946000	0.659910	0.735081	0.088932	0.978000	0.652890	0.742822
	$\alpha$	0.226696	0.943000	0.701331	0.928026	0.221161	0.943000	0.693137	0.914928
60	$\beta$	0.252641	0.948000	0.594624	0.847264	0.245972	0.946000	0.583608	0.825979
	*R*	0.061464	0.950000	0.662214	0.727677	0.073096	0.986000	0.660697	0.733794
	$\alpha$	0.195480	0.954000	0.712813	0.908293	0.191084	0.954000	0.706900	0.897984
80	$\beta$	0.216924	0.946000	0.606010	0.822933	0.211718	0.945000	0.598172	0.809890
	*R*	0.052783	0.953000	0.671491	0.724274	0.062520	0.973000	0.666805	0.729325

**Table 6:** The AWs and CPs of ACIs and HPDs for $\alpha$, $\beta$ and *R* ($\theta =5$).

*k*	Para	ACI				HPD			
		AW	CP	Lower	Upper	AW	CP	Lower	Upper
	$\alpha$	0.395378	0.931000	0.653644	1.049022	0.381499	0.934000	0.628106	1.009605
20	$\beta$	0.447049	0.929000	0.544605	0.991114	0.428577	0.941000	0.510641	0.939218
	*R*	0.096424	0.933000	0.658152	0.754575	0.116282	0.971000	0.649664	0.765946
	$\alpha$	0.271060	0.954000	0.686975	0.958034	0.264687	0.942000	0.674285	0.939152
40	$\beta$	0.301792	0.949000	0.577627	0.879419	0.293844	0.942000	0.561463	0.855307
	*R*	0.066173	0.943000	0.676930	0.743103	0.078727	0.976000	0.671241	0.749968
	$\alpha$	0.220192	0.943000	0.704204	0.924396	0.251128	0.938000	0.696215	0.911343
60	$\beta$	0.244853	0.945000	0.597643	0.842496	0.238850	0.940000	0.587082	0.825931
	*R*	0.054012	0.948000	0.682536	0.736548	0.063738	0.981000	0.678156	0.741894
	$\alpha$	0.189752	0.950000	0.715076	0.904828	0.185847	0.945000	0.709417	0.859263
80	$\beta$	0.210136	0.944000	0.608495	0.818632	0.205774	0.946000	0.601116	0.808690
	*R*	0.046372	0.953000	0.687250	0.733622	0.054595	0.977000	0.683497	0.738092

As demonstrated in Tables 4,5,6, the CPs of both ACI and HPD credible intervals show no significant difference; however, the CP of ACI is closer to the target value of 95%. The AWs of both ACI and HPD credible intervals decrease as the sample size *k* increases, and the CPs of both methods converges towards 95%, indicating improved estimation precision. Furthermore, with higher values of the dependent parameters, these intervals exhibit a smaller AW.

## Real data analysis

In this section, a real data analysis is provided to illustrate the established results in section "Inference of model parameters and reliability", and this data set has been studied by Akgül^36^, which is available in the link https://data.ibb.gov.tr/en/dataset/istanbul-dam-occupany-rates-data. The data we used is the dam occupancy rate of Istanbul, Turkey. These values were obtained from the Istanbul Metropolitan Municipality open data portal, covering the years 2006 to 2020. For each year, the occupancy rates of September, October, November, and December were extracted. If multiple records were available for a given month, their average was used to represent that month’s value. The resulting dataset (Table 7) provides the basis for the following analysis.

Due to the combined impacts of climate change and population growth, the risk of drought has become a significant concern in Istanbul, particularly during the dry season from September to December. To evaluate the effectiveness of water resource management strategies during this critical period, we construct a reliability-inspired indicator using a 2-2 series-parallel system based on the dam occupancy rates for these four months. Specifically, the four months are divided into two pairs: September and December form one pair, and October and November form the other. Within each pair, the higher occupancy rate is taken to represent the stronger monthly performance, forming the parallel components of the system. The lower of the two pairwise maxima is then used to represent the overall indicator(*Z*), corresponding to a series connection between the two subsystems. As a reference, we use the average occupancy rate over the same four-month period from the previous year(*Y*). If this year’s indicator *Z* exceeds the historical average *Y*, the strategy is considered effective in enhancing drought resilience. The values of *Z* and *Y* are presented in Table 8. In constructing this table, a non-overlapping year-pair structure is adopted, where each year’s indicator *Z* is paired with the baseline *Y* from its immediately preceding year. This design avoids repeated use of the same monthly data across adjacent years, thereby reducing potential temporal dependence between the constructed *Z* values.

**Table 7:** The dam occupancy rate from September to December.

	2006	2008	2010	2012	2014	2016	2018	2020
September	0.616333	0.181747	0.681890	0.563903	0.165963	0.486073	0.580257	0.420393
October	0.565781	0.248903	0.650348	0.490523	0.232306	0.416548	0.532723	0.331742
November	0.603870	0.274510	0.722240	0.471123	0.378613	0.360290	0.486993	0.274740
December	0.578526	0.290826	0.757077	0.568581	0.519668	0.395029	0.698077	0.221232

**Table 8:** The data *Z* and *Y*.

	2005	2007	2009	2011	2013	2015	2017	2019
*Y*	0.569238	0.166388	0.854897	0.594208	0.451997	0.650593	0.562861	0.432093

	2006	2008	2010	2012	2014	2016	2018	2020
*Z*	0.603870	0.274510	0.722240	0.490523	0.378613	0.416548	0.532723	0.331742

Applying Pearson’s method, we obtained the correlation coefficient (0.8545) between data *Z* and *Y*. The *t*-test yielded test statistic (4.0293) with corresponding *p*-value (0.0069), indicating a significant dependence between *Z* and *Y*. Table 9 presents the results of the goodness-of-fit test for the Clayton, Gumbel, and Frank copula applied to *Z* and *Y*. Among the copula tested, the Clayton copula, with a test statistic (0.0547) and *p*-value(0.1040), most accurately characterizes the dependence structure between *Z* and *Y*.

**Table 9:** Goodness-of-fit test for copula.

	Clayton	Gumbel	Frank
statistic	**0.0547**	0.0616	0.0704
*p*-value	**0.1040**	0.0545	0.0248

**Fig. 4:**
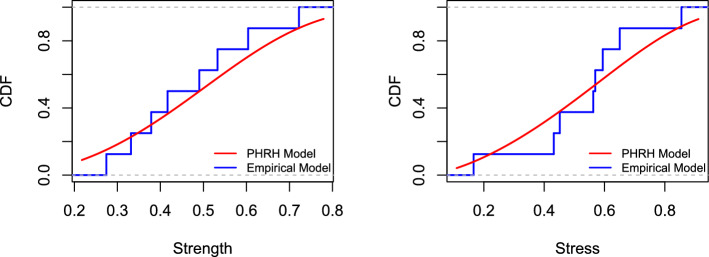
Plots of the empirical distribution and the fitted distribution of real data sets.

Initially, it is essential to verify whether the PRHR model is applicable for analyzing these data sets. The estimated parameters for *Z* and *Y*, along with the Kolmogorov-Smirnov statistics, corresponding *p*-values, Anderson–Darling (AD) statistics, and their *p*-values are detailed in Table 10. These results confirm that the PRHR model adequately fits the data sets. Furthermore, visual comparisons in Fig. 4 confirm that the PRHR model provides a good fit to both data sets.

**Table 10:** Results of the K-S test and AD test for the real data.

	$\hat{\alpha }$	$\hat{\beta }$	KS	*p*-value	AD	*p*-value
*Z*	0.2294	-	0.1720	0.9415	0.3672	0.8775
*Y*	-	0.3143	0.2296	0.7127	0.5034	0.7381

Based on real data and the base distribution $F(t)= 1- \exp (-2.5t^{5})$, the MLEs and Bayesian estimators and 95% ACIs and HPD CIs of $\alpha$, $\beta$ and *R* are given in Table 11.

**Table 11:** Estimate results of $\alpha , \beta$ and *R* based on real data.

Parameters	Estimator				CI	
	MLE	$\hbox {Bayes}_{SEL}$	$\hbox {Bayes}_{LL}$	$\hbox {Bayes}_{GEL}$	ACI	HPD
$\hat{\alpha }$	0.2294	0.2196	0.2195	0.2149	(0.1583, 0.3324)	(0.1353, 0.3019)
$\hat{\beta }$	0.3143	0.3008	0.3005	0.2882	(0.1871, 0.5280)	(0.1411, 0.4569)
$\hat{R}$	0.4202	0.4185	0.4209	0.4386	(0.1574, 0.6830)	(0.1198, 0.6452)

## Conclusion

When the stress and strength variables follow the PRHR model, and the dependence between stress and strength is characterized by the Clayton copula, we have addressed the estimation of dependent stress-strength reliability for series-parallel systems. Both classical and Bayesian statistical inference methods are utilized to estimate the model parameters and system reliability. We have established the MLE for the model parameters and system reliability, along with an improved approximate confidence interval based on Fisher information. Bayesian estimation is conducted using the flexible Gamma-Beta prior distribution under various loss functions, and the HPD interval is obtained through the Metropolis-Hastings algorithm. The Bayesian estimation based on the GEL function demonstrates superior performance, with a smaller AB compared to the MLE. In terms of interval estimation, the CP of ACI is closer to the target value of 95%, outperforming the HPD credible intervals. Monte Carlo simulations are performed to evaluate and compare the performance of the proposed estimation methods. Additionally, we have demonstrated the applicability of our approach by analyzing a real data set, specifically the general dam occupancy rate of Istanbul. The results confirm the effectiveness of our methods in providing reliable and accurate estimates for multicomponent dependent stress-strength reliability in series-parallel systems.

Since sample censoring is common in many practical problems, the inference of stress-strength reliability based on Type-I or Type-II censored samples presents an interesting challenge and will be explored in future research. As suggested by one of the reviewers, the scenario in which the baseline distribution in the PRHR model involves unknown parameters will be explored as a potential topic for future research.

## Data Availability

The datasets analysed during the current study are available in the Dam Occupancy Data repository, https://data.ibb.gov.tr/en/dataset/istanbul-dam-occupany-rates-data.
